# Contrasting Diversity and Composition of Human Colostrum Microbiota in a Maternal Cohort With Different Ethnic Origins but Shared Physical Geography (Island Scale)

**DOI:** 10.3389/fmicb.2022.934232

**Published:** 2022-07-12

**Authors:** Wanying Xie, Huimin Zhang, Yongqing Ni, Yunhua Peng

**Affiliations:** ^1^Department of Obstetrics and Gynecology, Hainan Medical University, Haikou, China; ^2^School of Food Science and Technology, Shihezi University, Xinjiang, China; ^3^The First Affiliated Hospital of Hainan Medical University, Haikou, China

**Keywords:** 16S rRNA gene, ethnicity, colostrum microbiome, Island scale, diversity

## Abstract

Colostrum represents an important source for the transfer of important commensal bacteria from mother to newborn and has a strong impact on the newborn’s health after birth. However, the composition of the colostrum microbiome is highly heterogeneous due to geographic factors and ethnicity (maternal, cultural, and subsistence factors). By analyzing the colostrum 16S rRNA gene full-length sequencing dataset in 97 healthy mothers (60 from Han, 37 from Li) from the Hainan island of China, we showed that the ethnic differences of the colostrum microbiome in a maternal cohort with different ethnic origins shared physical geography. Results indicated that the richness of microbial community in colostrum of Han women was higher than that of Li women, but there was no significant difference in Shannon index and invsimpson index between the two groups. Visualization analysis based on the distance showed an obvious ethnicity-associated structural segregation of colostrum microbiota. The relative abundance of *Firmicutes* was higher in the microbiota of the Han group than in Li’s, while *Proteobacteria* was on the contrary. At the genus level, the most dominant members of the Han and Li ethnic groups were *Acinetobacter* and *Cupriavidus*, two common environmental bacteria, respectively, although skin-derived *Staphylococcus* and *Streptococcus* were still subdominant taxa. *Cupriavidus lacunae* was the most dominant species in the Li group, accounting for 26.10% of the total bacterial community, but only 3.43% for the Han group with the most dominant *Staphylococcus petrasii* (25.54%), indicating that human colostrum microbiome was more susceptible to local living environmental factors. Hence, the ethnic origin of individuals may be an important factor to consider in human milk microbiome research and its potential clinical significance during the perinatal period in ethnic-diverse societies, even within a small geographic scale.

## Introduction

Colostrum is the first milk sucked by a baby after birth, and it is usually produced within 4–5 days after the mother has given birth (Fernández and Rodríguez, 2020; Stinson et al., 2021). Human milk (HM) contains the considerable beneficial nutrients and biologically active factors (Andreas et al., 2015; Williams et al., 2017), and it is universally considered the optimal source of nutrition for almost all healthy infants (Hunt et al., 2011; Lloyd-Price et al., 2016). In the meantime, increasing evidence shows that HM contains a diverse range of microbes (Oikonomou et al., 2020; Zimmermann and Curtis, 2020), which has important health implications for both mothers (mammary gland health) and infants (protection from diarrheal and respiratory diseases) (Lyons et al., 2020). Studies have shown that the relative abundance of potentially beneficial microbiota *Lactobacillus* and *Bifidobacterium* in exclusively breastfed infants is significantly higher than that in mixed-fed and formula-fed infants (Fehr et al., 2020; Lyons et al., 2020). Therefore, breastfeeding is one of the most optimum feeding regimes for newborn infants.

The maternal gut is thought to be the most important source of bacteria that are detectable in HM (*via* an entero-mammary pathway). However, it is incredible that more than 1,300 species and 3,500 operational taxonomic units of bacteria have been reported to be present in HM, even implying that the bacterial diversity in breast milk appears to be higher than in infant or maternal feces (Zimmermann and Curtis, 2020). Obviously, not all the bacteria detected in breast milk are considered inherent inhabitants of the mammary gland, and instead, a fairly large number of them come from environmental exposure (skin microbiota of the mother and the oral cavity of the infant), leading to significant differences between ethnic groups and/or even inter-individuals (Pannaraj et al., 2017). According to existing data, colostrum is characterized by higher diversity and more significant disparity in microbiome composition, compared with mature milk.

Depending on the source of bacteria, multiple factors could contribute to shaping the milk microbiota (Andreas et al., 2015; Zimmermann and Curtis, 2020). On the one hand, the microbial composition and diversity of breast milk may be influenced by maternal characteristics, including ethnicity (Deschasaux et al., 2018; Xu et al., 2020; Shafiee et al., 2022), pregnancy age, body mass index (BMI) (Cabrera-Rubio et al., 2012), mode of delivery (Cabrera-Rubio et al., 2012), parity, and intake of intrapartum antibiotics or probiotics (de Andrade et al., 2021). Meanwhile, several studies have reported differences in the microbiota composition of HM in different geographic locations, just as they do in the human skin metagenome (Gupta et al., 2017). Geography is an ensemble of multiple factors responsible for geography-based alterations in microbiota, including environmental (temperature, humidity, and altitude), population genetic, and cultural factors. In terms of microbiome studies, host surface-associated microbiomes could respond strongly to variations in environmental factors (Woodhams et al., 2020). Therefore, it is reasonable to speculate that bioclimatic factors would shape the composition of the human breast milk microbiome by exerting a force over skin microorganisms.

Modern molecular techniques, especially next-generation sequencing (NGS), are a more sensitive and less biased analytical method than the culture-based method and have been adopted for characterization of the composition and diversity of the human microbiome by using the 16S rRNA gene (Bardanzellu et al., 2017). To date, most of the studies utilized a shorter variation region of the 16S rRNA gene to profile human breast milk microbiota, such as the 16s rRNA gene V4 or V4-V5 region (Kumar et al., 2016; Ojo-Okunola et al., 2018) and the V1-V3 region (Williams et al., 2017). Due to the drawback of the short reading length, the composition of breast milk microbes cannot be exactly documented (Jost et al., 2012; Walker et al., 2015). By contrast, 16S rRNA gene full-length amplicon sequencing could achieve more accurate representation by providing species-level microbiome data (Lopez Leyva et al., 2021).

Human Milk presents an interplay between a mother and her infant from an evolutionary perspective. The various components of colostrum have a great impact on the newborn’s health after birth. Among them, the human milk oligosaccharides (HMOs) are thought to play a role in preventing pathogenic bacterial adhesion and orchestrating the development of the microbiota (Cheema et al., 2022; Sprenger et al., 2022). Particularly, bacteria in colostrum can stimulate the anti-inflammatory response by stimulating the production of specific cytokines, gradually promoting the maturation of the newborn’s immune system, although most of them might not be residents of infant gut microbiota. However, the high heterogeneity is characteristic of the composition of the colostrum microbiome depending on geographical and ethnic variations (maternal, cultural, and subsistence factors) (Gupta et al., 2017). So, parsing the appreciable disparity of colostrum microbiome between sub-populations in the same locality helps understand the clinical significance of breast milk microbiota in the perinatal period.

In the present study, we analyzed the NGS datasets of bacterial 16S rRNA full-length gene of colostrum samples in a maternal cohort containing two different ethnic groups, which included 97 healthy mothers (60 from Han and 37 from Li) from Hainan Island, the southernmost province in China. Historically, Li ethnic group is an indigenous people who live mostly in rural areas; Most of the Han Chinese are immigrants and live in cities or towns. We aimed to gain insight into the colostrum microbiome patterns of different sub-populations with shared physical climate in the narrow region (Island scale) and to assess how ethnicity (maternal, cultural factors, and subsistence) influences microbiota in the breast milk of healthy mothers.

## Materials and Methods

### Sample Collection

In this study, a total of 97 mothers (18–41 years old, with an average age of 28 years) after childbirth were recruited. The above volunteers all lived in Hainan for a long time. Among them, 37 mothers are of Li nationality and 60 mothers of Han nationality. Demographic data about the volunteer mothers’ BMI, delivery mode, and the use of antibiotics and probiotics during pregnancy were summarized in Table 1. When collecting samples, they have informed and signed an informed consent form for themselves and their family members. In addition, this study has also been approved by the ethics committee of Shihezi University.

**TABLE 1:** Demographic characteristics of the mothers in the study population.

Characteristics and demographic data	Values or (%)
**Ethnicity**	
Li	37 (38.1%)
Han	60 (61.9%)
**Age**	
18–25	21 (21.6%)
26–35	67 (69.1%)
36–45	9 (9.3%)
**Maternal BMI condition**	
Normal (18.0–23.9)	24 (24.7%)
Slightly fat (24.0–26.9)	28 (28.8%)
Obesity (27.0–29.9)	30 (30.9%)
Severe obesity (≥ 30.0)	16 (15.6%)
**Lifestyle**	
Farmer	51 (52.6%)
Urban	46 (47.4%)
**Delivery mode**	
Vaginal	70 (72.2%)
Cesarean	27 (27.8%)
**Intrapartum antibiotics**	
Received	18 (18.5%)
Not received	79 (81.5%)
**Parity**	
0	38 (39.2%)
1	46 (47.4%)
2–3	13 (13.4%)

Colostrum samples were collected into sterile tubes by manual expression using sterile gloves after nipples and areolas were cleaned with a swab soaked in sterile water or saline (Rodriguez-Cruz et al., 2020); the first 1–2 mL of milk was discarded to avoid contamination from the environment as much as possible (Douglas et al., 2020). Then, 5–15 mL of milk was collected and was immediately frozen and stored at −80°C until DNA extraction.

### DNA Extraction

FastPure Bacteria DNA Isolation Mini Kit (Vazyme, Nanjing, China) was used for the extraction of breast milk DNA with slight modification and combined with the glass bead beating method (Cheema et al., 2021; Lyons et al., 2021). About 1 mL of breast milk was centrifuged at 12,000 rpm (∼13,400 × *g*) for 10 min at 4°C, and the fat was removed with a sterile cotton swab and the supernatant was discarded (Ojo-Okunola et al., 2020). Add lysozyme (100 mg/mL) to the centrifuge tube and bath at 37°C for 30 min to achieve the purpose of enzymatic hydrolysis; then add 0.25 g zirconium beads (0.1 mm) and use a cell tissue disruptor to physically break the cell wall. After the fragmentation is completed, add 250 μL of Buffer GB, shake and mix, and incubate at 70°C for 10 min; add 4 μL RNase A to the digestion solution and heat at 65°C for 10 min to remove the RNA and obtain pure DNA as much as possible; add Proteinase K (20 mg/mL) to the sample and incubate at 58°C for 30 min to make it fully active (Hunt et al., 2011); then follow the steps in the instructions for column purification. Each DNA pellet was resuspended in 50–100 μL of Elution Buffer.

The DNA was quantified using a NanoDrop ND-2000 spectrophotometer (NanoDrop Technologies, Wilmington, DE, United States), and the remaining DNA was stored in a refrigerator at −20°C until the next step.

### The 16S rRNA Full-Length Amplicon Sequencing

The microbial library construction and sequencing of the total DNA of 97 colostrum samples were completed by Shanghai Personal Biotechnology Co., Ltd. (Shanghai, China). PCR amplification of full-length 16S rRNA gene was performed using a forward primer PB_16s_Bac-F (5′-AGAGTTTGATCMTGGCTCAG-3′) and a reverse primer PB_16s_Bac-R (5′-ACCTTGTTACGACTT-3′). To distinguish each sample, a unique barcode was added to each sample for specific amplification. The PCR amplification system was 25 μL, including 5 × reaction buffer 5 μL, 5 × GC buffer 5 μL, dNTP (2.5 mM) 2 μL, barcoded primers with forward primer (10 μM) 1 μL, reverse primer (10 μM) 1 μL, DNA template 2 μL, double distilled water (ddH_2_O) 8.75 μL, and Q5 DNA Polymerase 0.25 μL. The PCR conditions were as follows: 98°C for 2 min, 30 cycles at 98°C for 15 s, 55°C for 30 s, and 72°C for 30 s, and final extension at 72°C for 5 min. Sequencing was performed with PacBio Sequel sequencer.

### Raw Sequence Analysis

The original data files are converted into FASTQ format files and saved by using CCS v 4.0.0 [Generate Highly Accurate Single-Molecule Consensus Reads (HiFi Reads) software]. Use Perl^1^ script to divide the barcode sequence at both ends of the sequence, remove the barcode, and then transpose the reverse complementary sequence to the forward direction according to the primer sequence. Raw sequences were processed by using a pipeline combining USEARCH 11.0 Linux 64-bit and QIIME2. High-quality reads, as selected using the default values in USEARCH, were binned into amplicon sequence variants (ASVs) according to the denoising (error-correcting) Illumina amplicon reads using Unoise3, through an open-reference strategy. Taxonomic identification of ASVs for the sequences was assigned using the Naive Bayes classifier of the Ribosomal Database Project (RDP) against the Greengenes database and generated the feature table for subsequent analysis.

### Diversity Analysis and Significant Difference Analysis Between Ethnic Groups

Alpha diversity indices were calculated in QIIME2 from rarefied samples using the Chao1 and ACE indexes for richness, and the Shannon and invsimpson indexes for diversity, and statistics and the difference check box plot were performed using the personalbio genescloud platform.^2^ Beta diversity was calculated using Bray–Curtis distance, and principal coordinates analysis (PCoA) was performed. VENN analyses were also conducted using the R package Statistical analyses between different groups and were analyzed using ANOVA (Liu et al., 2021). Cytoscape_v3.8.2 was used to draw the network diagram. Mann–Whitney U-test was used for diversity and taxonomic comparisons between groups at different levels (phylum, genus, and species). Based on the standardized matrix and grouping information, STAMP 2.1.3 was used to analyze different genus and species. Linear discriminant analysis (LDA) effect size (LEfSe) analysis was performed at http://www.ehbio.com/Cloud_Platform/front (Liu et al., 2021). IBM SPSS statistical 26 was used to calculate the *p*-value. The *p* < 0.05 was considered statistically significant.

## Results

### Description of the Study Population

The socio-demographic characteristics of Li (*n* = 37) and Han’s mothers (*n* = 60) are summarized in Table 1. The mean maternal age in both groups was 28 years old. The average BMI was 27.1 and 25.5 for women in the Li and Han ethnic groups, respectively.

Table 2 showed the significant analysis results of the interaction between ethnic and other mother-related factors. BMI was significantly correlated with ethnic factors and the same as the mode of delivery (*p* < 0.05). There were extremely significant differences between the Li and Han groups in the mother’s lifestyle and the use of intrapartum antibiotics (Chi-square, both *p* = 0.001). There were no significant differences in maternal age and parity between Li and Han ethnic groups (all *p* > 0.05).

**TABLE 2:** Socio-demographic characteristics of subjects.

Characteristics	Li (*n* = 37)	Han (*n* = 60)	*p*-value
Age (mean ± SD, range)^1^	27.9 ± 5.7 (18–41)	29.2 ± 4.1 (21–41)	0.267
18–25	11 (29.7%)	10 (16.7%)	
26–35	21 (56.8%)	46 (76.7%)	
36–45	5 (13.5%)	4 (6.7%)	
Maternal BMI condition (mean ± SD, range)^1^	25.5 ± 3.1 (19–31)	27.1 ± 3.4 (21.5–35)	0.023*
Normal weight (18.0–23.9)	11 (29.7%)	13 (21.7%)	
Slightly fat (24.0–26.9)	15 (40.5%)	16 (26.7%)	
Obesity (27.0–29.9)	7 (18.9%)	19 (31.7%)	
Severe obesity (≥ 30.0)	4 (10.8%)	12 (20.0%)	
Lifestyle^2^			0.001***
Farmer	37 (100%)	13 (21.7%)	
Urban	0 (0%)	47 (78.3%)	
Delivery mode^2^			0.009**
Vaginal delivery	32 (86.5%)	37 (61.7%)	
C-section	5 (13.5%)	23 (38.3%)	
Intrapartum antibiotics^2^			0.001***
Received	0 (0%)	18 (30.0%)	
Not received	37 (100%)	42 (70.0%)	
Parity^2^			0.065
0	10 (27.0%)	28 (46.7%)	
1	19 (51.4%)	27 (45.0%)	
2–3	8 (21.6%)	5 (8.3%)	

^1^t-test; ^2^Chi-square. *p < 0.05, **p < 0.01, ***p < 0.001.

At the same time, we conducted a multivariate analysis of variance. The results of the analysis are shown in Supplementary Table 1. We did not discuss the effects between the two groups in detail when considering the distribution of the sample and the effects of the statistical test.

### DNA Sequencing and Filtering

A total of 859,638 16S rRNA raw reads were generated from the 97 samples. After filtering low-quality sequences, 859,345 filtered sequences were retained with lengths measuring 1,200–1,500 bp. All 859,345 high-quality sequences were clustered into ASVs at 100% sequence similarity using Quantitative Insights Into Microbial Ecology (QIIME2) software. An average number of high-quality sequences in each sample reached 17,543 and a total of 789 ASVs were discovered in 97 samples.

### Diversities of Bacterial Communities of Colostrum Across Two Ethnic Groups

**FIGURE 1:**
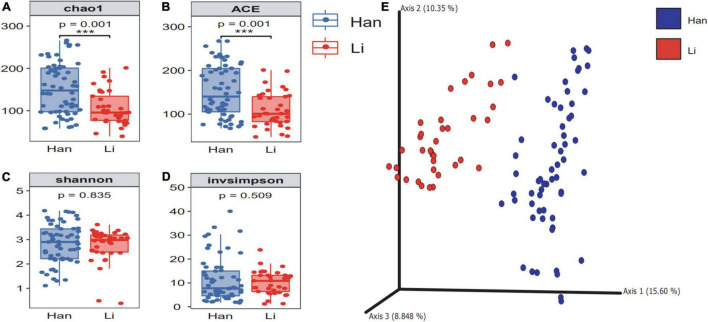
The α diversity and β diversity of colostrum microbiota of two ethnic groups. The difference check box plot shows the alpha diversity between the Li and Han ethnic groups. chao1 **(A)** and ACE **(B)** can reflect the abundance, and Shannon **(C)** and invsimpson **(D)** can reflect the diversity between Li and Han’s mothers analyzed by 16S rRNA gene sequencing. **(E)** Principal coordinate analysis plot of Bray–Curtis distance.

According to the number of ASVs, the alpha diversity of microflora in colostrum was calculated under different maternal-related factor grouping (Supplementary Table 2). Results showed that ethnicity, age, and lifestyle had a significant effect on the microbial richness of colostrum, among which the ethnic factor had the most significant impact on the richness. Then, when we grouped by ethnicity, the chao1 of colostrum microbiome in Han’s mothers (151.54 ± 60.86) was significantly (*p* = 0.001) higher than the index of colostrum in Li’s mothers (106.75 ± 40.06) (Figure 1A), and the same trend was observed in the index of ace (Li 108.99 ± 39.46 vs. Han 152.65 ± 59.29) (Figure 1B). However, we did not find a significant difference in the Shannon and invsimpson index (Figures 1C,D). (Li 2.76 ± 0.69 vs. Han 2.82 ± 0.81) (Li 10.15 ± 4.97 vs. Han 11.15 ± 9.14). To investigate the taxonomic structural distinctiveness of colostrum microbial communities between Li and Han Ethnic Groups, beta-diversity analysis was conducted based on the Bray–Curtis distance (Figure 1E). There is an obvious structural separation between the two ethnic groups.

### Impact of Ethnic and Delivery Mode on Microbial Diversity in Colostrum

**FIGURE 2:**
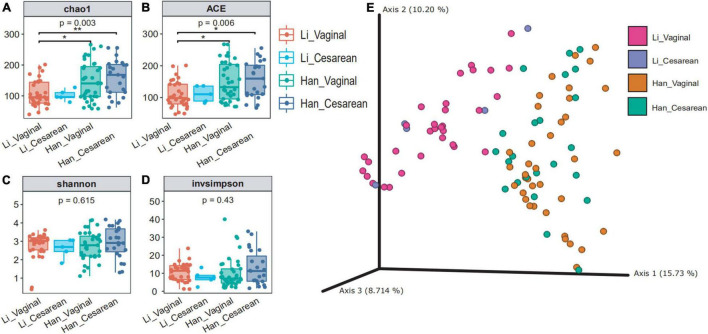
Colostrum microbial diversity in China’s Hainan cohort grouped by ethnicity (Han/Li) and mode of delivery (cesarean/vaginal). chao1 **(A)** and ACE **(B)** reflected the abundance of the four groups (Li_Vaginal and Li_Cesarean and Han_Vaginal and Han_Cesarean), and Shannon **(C)** and invsimpson **(D)** reflected the diversity. **(E)** Bray–Curtis distance of the 97 samples using the full set of ASVs. The percentage of the variation explained by the plotted principal coordinates (PCs) was shown in parentheses.

When we grouped all the data by the mode of delivery (vaginal vs. cesarean), the diversity of the cesarean group was higher than that of the vaginal group (Supplementary Table 2). Based on the ethnicity (Han vs. Li) and the mode of delivery (vaginal vs. cesarean), 97 samples were divided into four groups (Han_vaginal and Han_cesarean and Li_vaginal and Li_cesarean). The chao1 and ace indexes showed that there were significant differences between the colostrum microbial α-diversity of the mothers of the Li_vaginal group and the Han_vaginal group (Figures 2A,B). When we consider the richness of microbiota, the results showed that the richness indexes (chao1 and ace) of the Han_cesarean group were the highest, followed by the Han_vaginal group. Different from the Han ethnic group, the diversity index of the Li_vaginal group was higher than that of the Li_cesarean group. The Shannon and invsimpson index showed that there was no significant difference among the four groups (Figures 2C,D). β-diversity analysis showed that the two groups of the same ethnic group among the four groups were gathered, that is, the Han_vaginal group and the Han_cesarean group were clustered together and the Li_vaginal group and the Li-cesarean group were gathered (Figure 2E). In conclusion, PCA showed that there was an obvious division in the diversity of colostrum microbes between Li and Han ethnic groups regardless of the grouping of delivery modes.

### Colostrum Microbiota Compositional Analysis

We profiled the bacterial composition of colostrum microbiota between different groups at the level of phylum, genus, and species. Bacterial taxa with a relative abundance of less than 1% in individual samples were categorized into the “others” group.

The results of the phylum level show that the most dominant phyla in Li colostrum is *Proteobacteria* (66.5%), followed by *Firmicutes* (29.5%). Interestingly, the results of the dominant phylum in the microbial composition of breast milk in the Han population are completely opposite; *Firmicutes* is the most dominant phylum with a relative abundance of 46.5% and *Proteobacteria* with a relative abundance of 43.7% (Table 3 and Supplementary Figure 1A). When analyzing at the genus level, *Cupriavidus* (26.28%), *Staphylococcus* (17.36%), and *Streptococcus* (13.11%) were relatively abundance genera in the Li population cohort; while in Han colostrum, *Acinetobacter* (28.72%), *Staphylococcus* (28.38%), and *Streptococcus* (9.45%) were dominant genera. Table 3 and Supplementary Figure 1C showed that the microbial components of colostrum between the two ethnic groups are more abundant and diverse at the species level. Among the Li population, species with a relative abundance above 1% were *Cupriavidus lacunae* (26.10%), *Staphylococcus petrasii* (16.60%), *Enterobacter hormaechei* (7.83%), *Streptococcus himalayensis* (5.50%), and *Streptococcus panodentis* (1.09%). In the Han group, there were eight species with an abundance of more than 1%, namely *Staphylococcus petrasii* (25.54%), *Streptococcus himalayensis* (4.37%), *Citroniella saccharovorans* (4.11%), *Cupriavidus lacunae* (3.43%), *Enterobacter hormaechei* (2.67%), *Staphylococcus pseudoxylosus* (1.96%), *Streptococcus panodentis* (1.93%), and *Klebsiella aerogenes* (1.01%).

**TABLE 3:** Associations between maternal characteristics and top 4 phyla, 11 genera, and 11 species.

	All	Han	Li	Han-vaginal	Han-cesarean	Li-vaginal	Li-cesarean
**Phylum**	%						
*Firmicutes*	45.90	47.10	34.43	45.54	49.62	34.54	33.76
*Proteobacteria*	36.50	43.02	61.54	46.12	38.03	61.28	63.18
*Actinobacteria*	9.90	3.46	2.61	2.70	4.67	2.69	2.11
*Bacteroidetes*	5.20	0.58	1.22	0.34	0.96	1.27	0.88
**Genus**							
*Streptococcus*	17.70	9.45	13.11	10.36	7.99	12.88	14.58
*Acinetobacter*	10.00	28.72	10.41	30.08	26.54	10.59	9.26
*Staphylococcus*	9.60	28.38	17.36	27.54	29.75	17.52	16.35
*Pseudomonas*	3.70	0.02	0.85	0.02	0.03	0.88	0.65
*Corynebacterium*	2.90	0.55	0.33	0.16	1.19	0.27	0.69
*Enterobacter*	2.40	3.25	12.13	4.69	0.92	12.07	12.51
*Prevotella*	2.30	0.04	0.97	0.05	0.03	0.99	0.80
*Cutibacterium*	1.10	1.42	1.82	1.08	1.97	1.99	1.22
*Cupriavidus*	0.90	3.74	26.28	2.57	5.61	25.74	29.72
*Rhodopseudomonas*	0.40	0.17	0.91	0.11	0.27	0.97	0.51
*Paucibacter*	0.30	0.64	4.17	0.38	1.67	4.16	4.24
**Species**							
*Streptococcus himalayensis*	9.90	4.37	5.50	3.62	5.56	5.78	3.69
*Staphylococcus petrasii*	7.00	25.54	16.06	24.97	26.45	16.15	15.49
*Streptococcus panodentis*	2.40	1.93	1.09	2.61	0.83	0.66	3.84
*Enterobacter hormaechei*	1.80	2.67	7.83	3.91	0.69	7.73	8.49
*Citroniella saccharovorans*	1.60	4.11	0.04	5.62	1.68	0.04	0.05
*Klebsiella aerogenes*	1.60	1.01	0.18	1.50	0.23	0.20	0.01
*Staphylococcus pseudoxylosus*	1.50	1.96	0.88	1.59	2.56	0.90	0.73
*Escherichia/Shigella coli*	1.50	0.80	0.07	1.16	0.21	0.08	0.07
*Acinetobacter pseudolwoffii*	1.10	0.19	0.02	0.18	0.20	0.02	0.05
*Acinetobacter modestus*	1.60	0.09	0.51	0.04	0.18	0.41	1.16
*Cupriavidus lacunae*	0.60	3.43	26.10	2.30	5.25	25.57	29.50
Unassigned	30.40	18.40	11.54	20.14	15.59	11.82	9.74

### Core Microbiota Analysis of Colostrum Based on Amplicon Sequence Variant Level

**FIGURE 3:**
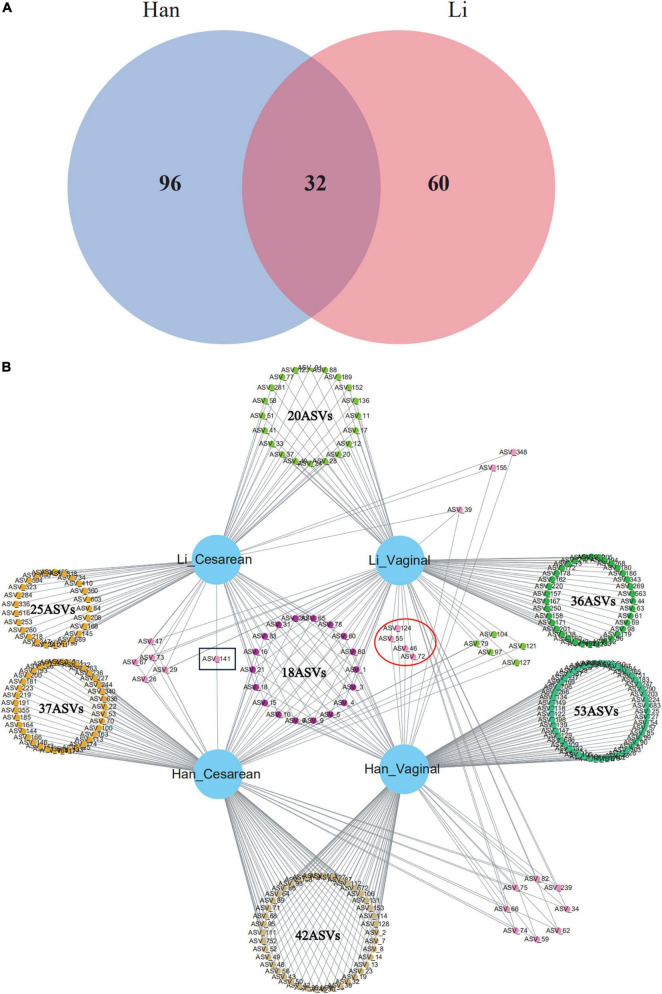
Venn diagram of core ASVs (or known bacterial species) sharing among **(A)** Han and Li ethnic groups or **(B)** ethnic and delivery mode groups (Li_Vaginal and Li_Cesarean and Han_Vaginal and Han_Cesarean).

The core composition of colostrum bacteria and specific ASVs or species with a relative abundance of more than 0.1% were screened from 97 samples. The overlapping areas of the circles in the Venn diagram represent the core microbiome, which is generally defined as a shared group of microbiome members from similar habitats. As shown in the Venn diagram, a total of 32 ASVs were observed as common ASVs, 96 ASVs in the Han ethnic group, and 60 ASVs in the Li ethnic group (Figure 3A). The 32 ASVs assigned to the eight-core genera were *Staphylococcus*, *Acinetobacter*, *Streptococcus*, *Cutibacterium*, *Cupriavidus*, *Enterobacter*, *Rhodopseudomonas*, and *Paucibacter*. Among the 16 ASVs belonging to *Staphylococcus*, 14 ASVs can be classified to the *Staphylococcus* species level: *Staphylococcus pseudoxylosus* (ASV_59 and ASV_83) and *Staphylococcus petrasii* (ASV_3, ASV_4, ASV_18, ASV_34, ASV_62, ASV_65, ASV_66, ASV_74, ASV_75, ASV_82, ASV_124, and ASV_282). The four ASVs of Acinetobacter were divided into two species: *Acinetobacter courvalinii* (ASV_5 and ASV_10) and *Acinetobacter oleivorans* (ASV_21 and ASV_78). Among the 3 ASVs belonging to *Streptococcus*, ASV_29 and ASV_72 were classified as *Streptococcus himalayensis*, while ASV_9 could not be classified to the species level. ASV_47 and ASV_60 both belonged to *Cutibacterium acnes.* In addition, *Cutibacterium modestum* (ASV_26) could also be detected in our study. Other ASVs that can be identified include *Cupriavidus lacunae* (ASV_1), *Cupriavidus nantongensis* (ASV_80), *Enterobacter bugandensis* (ASV_15), *Rhodopseudomonas boonkerdii* (ASV_31), and *Paucibacter oligotrophus* (ASV_6).

When considering the mode of delivery and the ethnic factor, network analysis revealed the total number of ASVs among the four groups (Han_Cesarean, Han_Vaginal, Li_Cesarean, and Li_Vaginal). There were 42 ASVs in Han_Cesarean and Han_Vaginal groups, belonging to 11 genera: *Acinetobacter*, *Staphylococcus*, *Enterococcus*, *Streptococcus*, *Thermus*, *Meiothermus*, *Diaphorobacter*, *Cutibacterium*, *Brucella*, *Chryseobacterium*, and *Brachybacterium.* In the Li_Cesarean and Li_Vaginal groups, 10 ASVs were identified as *Acinetobacter*, *Enterococcus*, and *Streptococcus*, another 10 ASVs belong to *Novosphingobium*, *Ruegeria*, *Agrobacterium*, *Phytobacter*, *Delftia*, *Serratia*, and *Cupriavidus*. Only ASV_141 (*Corynebacterium*) belongs to the Cesarean group; while the vaginal group had four specific ASVs, including *Streptococcus himalayensis* (ASV_46 and ASV_72) and *Staphylococcus petrasii* (ASV_55), and ASV_124 could only be assigned into the *Staphylococcus* (Figure 3B).

### Microbial Signatures in Different Ethnic Group Samples

**FIGURE 4:**
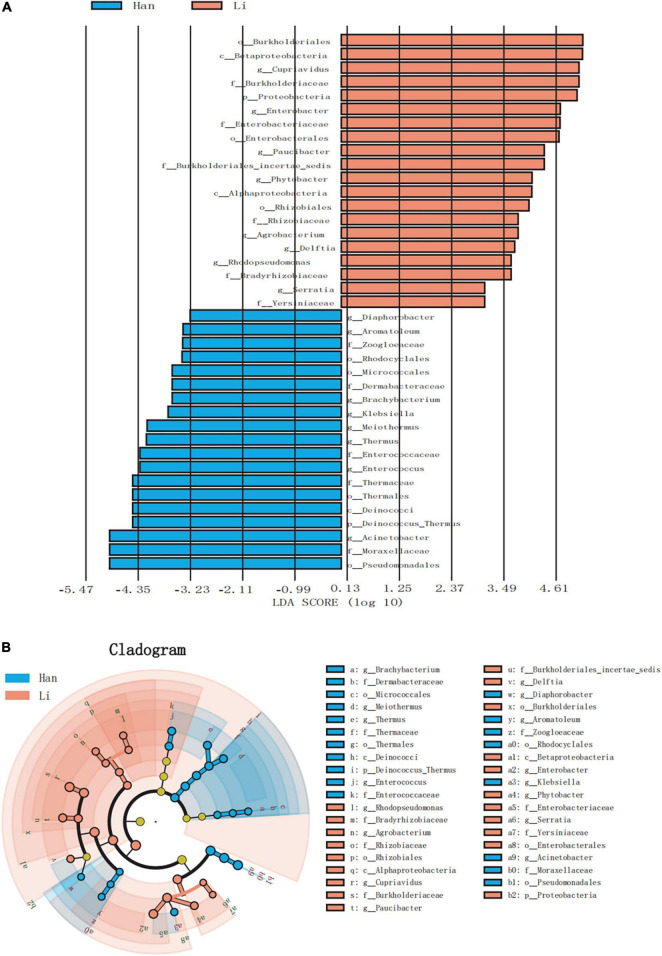
LEfSe based on bacterial communities in colostrum samples from two different ethnic groups. **(A)** Histogram of LDA value distribution (LDA score > 3.0). **(B)** LEfSe analysis evolutionary branch diagram.

Linear discriminant analysis effect size (LEfSe) analysis of ASVs, with an average relative abundance of>0.01%, was further conducted to detect microbial signatures in the colostrum of Han and Li ethnic groups. Figure 4A was a histogram of LDA value distribution, showing species with LDA Score greater than 3.0. The significant biomarkers in the entire Han and Li groups were mainly distributed in *Proteobacteria* and just several significant biomarkers were distributed in *Actinobacteria*, *Firmicutes*, and *Deinococcus_Thermus*. Analysis of the different species in Li people showed that they were all belonging to *Proteobacteria.* In the Li group, the LDA score was highest in *Burkholderiales*. The highest LDA score was found in the Acinetobacter of Han ethnicity. The LEfSe cladogram analysis revealed that 39 biomarkers of different classification levels were significantly different among the two groups (Figure 4B). Notably, we did not find any differences in biomarkers at the species level.

**FIGURE 5:**
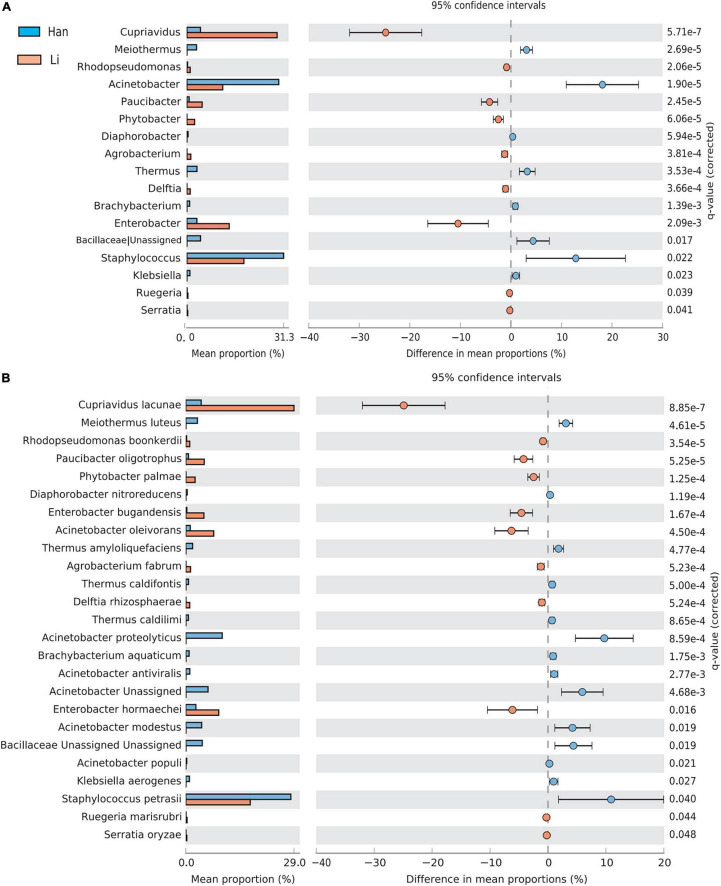
Analysis of the difference of taxa between groups at the genus **(A)** and species level **(B)**. Different colors represent different groups. The left figure in the picture shows the abundance ratio of different taxa in the two groups of samples, the middle shows the difference ratio of functional abundance in the 95% confidence interval, and the right value is the *p*-value.

Using the stamp software and the Benjamin FDR method, the extend-bar plot showed the difference between the two groups. Overall, 17 distinct genera and 25 distinct species were identified. *Cupriavidus* and *Enterobacter* were the two most significant genera in the Li ethnic group. *Staphylococcus* and *Actinobacteria* were the two most abundant genera in the Han ethnic group (Figure 5A). There were 11 species with higher abundance in the Li ethnic group and 14 species with higher abundance in the Han ethnic group (Figure 5B). *Cupriavidus lacunae* were the dominant species and the most distinct species in the Li ethnicity. In the meantime, *Enterobacter hormaechei* was rich in the Li group. The different species in the Han group were *Staphylococcus petrasii* and *Acinetobacter proteolyticus*.

### *Lactobacillaceae* and *Bifidobacterium* Profiles Identified by 16S rRNA Gene Sequencing

A total of 17 ASVs were identified as members of the *Lactobacillaceae, including Limosilactobacillus*, *Lactobacillus*, *Lacticaseibacillus*, *Levilactobacillus*, *Lactiplantibacillus*, and *Leuconostoc.* Among them, 13 ASVs were classified into the species, including *Leuconostoc mesenteroides* (ASV_164 and ASV_251), *Limosilactobacillus reuteri* (ASV_303, ASV_529, ASV_541, and ASV_637), *Lacticaseibacillus rhamnosus* (ASV_575), *Limosilactobacillus caviae* (ASV_385 and ASV_754), *Lactobacillus colini* (ASV_452), *Levilactobacillus bambusae* (ASV_419), and *Lactobacillus acidophilus* (ASV_447 and ASV_464). Only three samples (HNa10, HNa17, and HNa31) and ten samples (16.67%) from the Li and Han ethnic groups contained *Lacticaseibacillus rhamnosus*, respectively. As for *Limosilactobacillus reuteri*, the detection rate in Han ethnic group was 11.67% and the relative abundance was 0.05–0.10%; while in the Li ethnic group, we did not detect it.

In the present study, to obtain more accurate taxonomic results at the species level, the representative sequences of all five ASVs were identified as members of the *Bifidobacterium* genus. Due to the resolving power of the 16S rRNA gene in the identification of different bacteria species, four out of five ASVs were assigned to the *Bifidobacterium* species level: *Bifidobacterium castoris* (ASV_225), *Bifidobacterium longum* (ASV_461), and *Bifidobacterium scaligerum* (ASV_307 and ASV_647), respectively. ASV_607 can be only classified into the *Bifidobacterium* genus. In the Li ethnic group, *Bifidobacterium* did not detect in the colostrum of the Li ethnic group. While in the Han ethnic group, *Bifidobacterium* was detected in 35 samples, and the detection rate of *Bifidobacterium* was 60%. Overall, five samples (HNb15, HNb21 HNb27, HNb30, and HNb41) contained *B. castoris*, *B. longum*, and *B. scaligerum* with mean relative abundance ranging from 0.05 to 0.90%.

## Discussion

The breast milk microbiome can have a profound impact on human health by affecting the establishment of the neonatal intestinal flora and the development of the immune system (Fernández and Rodríguez, 2020; Yi and Kim, 2021). Some of the ethnic variations in microbiome structure have been attributed to differences in host genetics and innate/adaptive immunity, while in many other cases, maternal factors (age, BMI, mode of delivery, etc.), cultural features (diet, hygiene, environmental exposure, etc.), and subsistence factors overshadow genetics (Gupta et al., 2017). We focused on the ethnic group, which represents a highly diverse demographic character of the Chinese population (Table 1). A total of 97 Li and Han’s mothers, who lived in Hainan of China for a long time, were selected to collect their milk within 2–5 days after delivery and were used to compare the composition and diversity of colostrum microbiota.

Maternal factors, including pre-gestational BMI, age or mode of delivery, and other related factors have been proposed to influence colostrum microbiota composition (Zimmermann and Curtis, 2020). Our results showed that BMI and parity had no significant effect on the alpha-diversity of the colostrum microbial community (Supplementary Table 1). In terms of the delivery mode, our results reported a higher alpha-diversity in the colostrum of women delivered cesarean, which was consistent with the result of the study on the diversity of breast milk microbes in Taiwan and Mainland China (Li et al., 2017). However, the other two studies (84 and 393 participants, respectively) did not confirm this (Kumar et al., 2016; Moossavi et al., 2019). Previous studies also reported higher alpha-diversity and richness in the HM microbiota of women receiving intrapartum antibiotics (Hermansson et al., 2019). Our results suggest that intrapartum antibiotics had no significant influence on the diversity of colostrum microbiota. This could be attributed to the fact that women who have c-sections have a high rate of taking antibiotics during the perinatal period. It is now generally believed that the establishment of the human gut microbiota was influenced by the host’s genetics and diet and environmental exposure. The maternal gut is thought to be the most important source of bacteria in HM (*via* an entero-mammary pathway). So, the mother’s diet might influence the HM microbiota diversity by modifying the composition of the maternal gut microbiota (Biagi et al., 2017; Padilha et al., 2019).

Most studies reported consistently *Firmicutes* and *Proteobacteria* to be the most predominant phyla in both mature milk and colostrum (Sakwinska et al., 2016; Biagi et al., 2017). However, at the genus and species levels, there are significant differences in the composition of breast milk microbiome reported, with many genera found in less than 10% of studies. In addition, to distinguish stable and permanent microbiome members from the highly complex colostrum microbiota, which includes thousands of different species, we aimed to use the concept of the core microbiome (Lemanceau et al., 2017; Toju et al., 2018). Due to the resolution limits of DNA-based analyses, core microbiota had been predominantly defined using genus-level discrimination of a population. Nevertheless, a core microbiota of seven to nine bacterial genera was often proposed based on sample abundance (intestinal microbes, environmental microbes, and other related fields). In our study, using the 16S rRNA full-length amplicon technique, *Staphylococcus*, *Acinetobacter*, *Streptococcus*, *Cutibacterium*, *Cupriavidus*, *Enterobacter*, *Rhodopseudomonas, and Paucibacter* (Table 3) were selected as the core microbiota in 97 maternal colostrums from Hainan province according to the relative abundance of microbiota. Among them, *Cupriavidus*, being the most abundant genera in the Li ethnic group, was often found in soil (Estrada-de Los Santos et al., 2014), with isolates of *Cupriavidus lacunae* recovered in pond-side soil (Feng et al., 2019). Similarly, *Paucibacter* was also an environmental bacteria found in aquatic sediment.

Environmental exposure during the perinatal period (skin microbiota of the mother and the oral cavity of the infant) may be the main reason for broad differences in breast milk microbiome. Based on published data, colostrum displayed higher diversity and more significant disparity in microbiome composition than mature milk across geographically different populations, characterized by a higher prevalence of environmental bacteria. Indeed, the oral and skin microbiome are the next most diverse. In the case of the skin microbiome, rural and urban Chinese populations show variation in the abundance of some taxa, such as *Trabulsiella* and *Propionibacterium* (Gupta et al., 2017). Generally, host surface-associated microbiomes, such as skin microbiome, might respond strongly to variations in bioclimatic factors, thereby they may shape the composition of the human breast milk microbiome (Woodhams et al., 2020). In our study, Hainan Island of China has a tropical monsoon climate, characterized by hot and humid year-round, abundant rainfall. Interestingly, some thermotolerant environmental bacteria taxa first isolated from a hot spring, such as *Thermus amyloliquefaciens*, *Thermus caldifontis*, and *Meiothermus luteus* (Yu et al., 2015; Habib et al., 2017; Khan et al., 2017), were found in the most of Hainan colostrum samples. Moreover, another peculiarity from our data was the prevalence of other soil environmental bacteria in colostrum samples of Li’s mothers, such as the genus *Agrobacterium*. In fact, previous multiple studies showed that about half of dominant genera in colostrum belonged to environmental bacteria ubiquitous in soil and water, such as *Pseudomonas*, *Rhizobium*, *Acinetobacter, Alcaligenes*, and so on (Drago et al., 2017; Toscano et al., 2017). Consequently, some of the ethnic variations in the colostrum microbiome could be attributed to differences in cultural features/subsistence like diet, hygiene, and labor practice. This result is also consistent with the fact that most mothers the Li ethnic group recruited live mostly in rural areas and are engaged in farming. In other words, for most of the studies on breast milk microbiome, alcohol disinfection is not effective in preventing the detection of skin-associated microorganisms probably derived from exposed environment (soil and vegetation).

Based on the excessive presence of exogenous bacteria in breast milk, the most prevalent genera in breast milk microbiota were generally distinct from the most prevalent genera of the infant gut (Pannaraj et al., 2017; Fehr et al., 2020). However, there is a consensus that the first beneficial bacteria that enters the infant’s gut should be from the colostrum. Particularly, the commensal bacteria in colostrum could be selected to serve as seeds for newborns to initially establish a healthy gut microbiome. Several studies have shown that *Bifidobacterium* and *Lactobacillus* are highly present in the gut microbiota of infants and have been considered to be transmitted from mother to infants shortly after birth by breastfeeding, which can effectively avert irritable bowel syndrome and contribute to the development and balance of intestinal flora for infants (Yassour et al., 2018). Therefore, these potential probiotic commensal bacteria in colostrum are of particular concern, especially *Bifidobacterium, Lactobacillus*, and so on.

By reviewing the existing literature, the presence of *Bifidobacterium* and *Lactobacillus* was sporadically reported in a few colostrum samples or not at all (Gupta et al., 2017). In our study, about 48.5% of colostrum samples were retrieved using the 16S rRNA ASVs corresponding to family *Lactobacillaceae*, with about 0.28% mean relative abundance. According to NCBI BLAST homology search of 16S rRNA gene full-length sequencing, ASVs belonging to five new genera revised of the family *Lactobacillaceae* were retrieved, including *Limosilactobacillus*, *Lactobacillus*, *Lacticaseibacillus*, *Levilactobacillus*, *Lactiplantibacillus*, and *Leuconostoc* (Zheng et al., 2020). Taxa identified at the species level were *Limosilactobacillus reuteri*, *Limosilactobacillus caviae*, *Lactobacillus colini*, *Lactobacillus acidophilus*, *Lacticaseibacillus rhamnosus*, and *Levilactobacillus bambusae*. Surprisingly, *Bifidobacterium* did not detect in the colostrum of the Li ethnic group, but the detection rate was nearly 60% in the Han group, with mean relative abundance ranging from 0.05 to 0.9%. The *Bifidobacterium* species identified mainly included *B. longum*, *B. castoris, and B. scaligerum*. To date, most studies on breast milk using the NGS of different 16S variable gene regions reported their presence only at the taxonomic level of genus, with significantly different results. For example, in a study based on the V4 variable region of the 16S rRNA gene, the average relative abundances ranged from 0.1 to 1% for *Bifidobacterium* and from 0.1 to 0.3% for *Lactobacillus* (Padilha et al., 2019). In another study based on the V3-V4 region of the 16S rRNA gene, investigators reported around 2% average relative abundances of *Bifidobacterium* and *Lactobacillus* in the first weeks after delivery (Murphy et al., 2017). Intriguingly, an ASV affiliated with *Akkermansia* (as a kind of emerging candidate probiotics) was also detected in eight colostrum samples of the Han ethnic group (0.1%), belonging to *Akkermansia glycaniphila.* This was the first report that *Akkermansia* was detected in breast milk (Ouwerkerk et al., 2016).

To the best of our knowledge, this is the first study to reveal the composition and diversity of colostrum microbiome in different ethnic groups living in narrow geographical areas on an island scale. We tried to understand the influence of ethnicity on the colostrum microbiome in different sub-populations with shared physical geography by minimizing environmental factors. In fact, it is hard to tease out the relative contributions of geography and ethnicity to the breast milk microbiome, which are intertwined. Our study has limitation concerning the sample size and cohort populations. We will recruit multiple cohorts, including different cohorts of the same ethnic groups with different subsistence and living environments, and different ethnic groups sharing similar subsistence and living environments.

## Conclusion

In the present study, by analyzing the colostrum 16S rRNA gene full-length sequencing dataset in 97 healthy mothers (60 from Han, 37 from Li) from the Hainan island of China, we show the ethnic differences of the colostrum microbiome in a maternal cohort with shared physical geography. The analysis based on the Bray–Curtis distance showed an obvious ethnicity-associated structural segregation of colostrum microbiota. The human colostrum microbiome is more susceptible to local living environmental factors, although skin-derived *Staphylococcus* and *Streptococcus* are still subdominant taxa. Probably, environmental exposure during the perinatal period may be the main reason for broad differences in the colostrum microbiome. Consequently, colostrum displayed higher diversity and more significant disparity in microbiome composition than mature milk, characterized by a higher prevalence of environmental bacteria. In addition, despite the low relative abundance and presence of inter-population differences, the potential probiotic bacteria do exist in colostrum, especially *Bifidobacterium* and *Lactobacillus*. Our results suggest that the ethnic origin of individuals may be an important factor to consider in HM microbiome research and its potential clinical significance during the perinatal period in ethnic-diverse societies, despite a small geographic scale. Finally, further research is needed to tease out the relative contributions of geography and ethnicity to the breast milk microbiome.

## Data Availability Statement

The datasets presented in this study can be found in online repositories. The names of the repository/repositories and accession number(s) can be found below: NCBI - PRJNA845888, SRR19548162 - SRR19548258.

## Ethics Statement

The studies involving human participants were reviewed and approved by the Ethics Committee of the First Affiliated Hospital, Shihezi University School of Medicine (KJ2022-080-01). The patients/participants provided their written informed consent to participate in this study.

## Author Contributions

WX: resources, methodology, investigation, and writing. HZ: formal analysis, methodology, validation, and writing— original draft. YN: conceptualization and supervision. YP: conceptualization, project administration, and funding acquisition. All authors contributed to the article and approved the submitted version.

## Conflict of Interest

The authors declare that the research was conducted in the absence of any commercial or financial relationships that could be construed as a potential conflict of interest.

## Publisher’s Note

All claims expressed in this article are solely those of the authors and do not necessarily represent those of their affiliated organizations, or those of the publisher, the editors and the reviewers. Any product that may be evaluated in this article, or claim that may be made by its manufacturer, is not guaranteed or endorsed by the publisher.

## Footnotes

1
https://github.com/torognes/vsearch/wiki/VSEARCH-pipeline

2
https://www.genescloud.cn/login
