# Supplementary material for: Contrasting Diversity and Composition of Human Colostrum Microbiota in a Maternal Cohort With Different Ethnic Origins but Shared Physical Geography (Island Scale)
Source: Front Microbiol. 2022 Jul 12;13:934232. doi: 10.3389/fmicb.2022.934232 (PMC9315263; doi:10.3389/fmicb.2022.934232)
Supplement: Supplementary file 1 [file Data_Sheet_1.DOCX]

**Contrasting Diversity and Composition of** **Human Colostrum Microbiota** **in a**

**Maternal Cohort with Different Ethnic Origins but Shared** [**Physical**](D:/%E6%9C%89%E9%81%93%E8%AF%8D%E5%85%B8/Dict/8.10.3.0/resultui/html/index.html#/javascript:;)**Geography (Island Scale)**

*Wanying Xie^1^, Huimin Zhang^2^, Yongqing Ni^2^** and *Yunhua Peng^1,3^**

*^1^Hainan Medical University, Xueyuan Road, Longhua District, Haikou City, 571199, Hainan Province, People's Republic of China*

*^2^School of Food Science and Technology, Shihezi University,* *Fourth Nouth Ave., Shihezi, 832000, Xinjiang, People's Republic of China*

*^3^The First Affiliated Hospital of Hainan Medical University, Longhua Road, Haikou City, 570102, Hainan Province, People's Republic of China*

***Corresponding authors

Email addresses: Y. N.: [niyqlzu@sina.com](mailto:niyqlzu@sina.com); Y.P.: 2008pengyh@163.com

Tell: Y.N.:15299950600; Y.P.: 18919801677

**Contents**

**Supplementary Figures**3

Fig. S1 Bacterial taxonomic composition of colostrum from different groups in Hainan

3

**Supplementary Tables**

Table. S1 Multivariate redundance analyses showing association between ethnic, intrapartum antibiotics, mode of delivery, and other factors 4

Table. S2 Factors associated with colostrum microbiota α diversity.4

**Supplementary Figure**

**
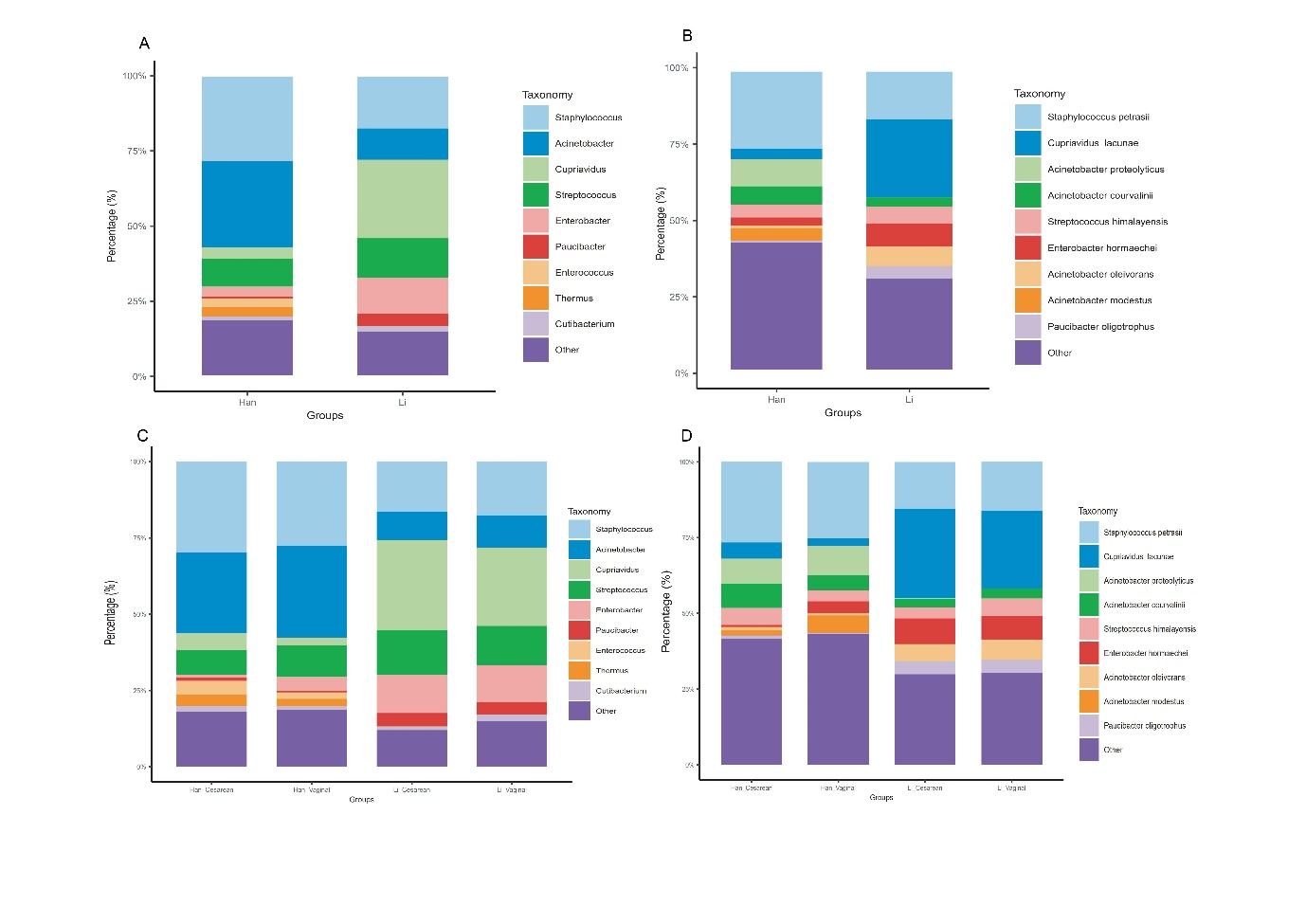
**

**FIG. S1** **Bacterial taxonomic composition of colostrum from different groups in Hainan**

The composition of colostrum microbes at the genus level and species level between the two ethnic groups was shown in (Fig. A) and (Fig. B), which inferred by polymerase chain reaction amplification and 16S rRNA gene full-length amplicon sequencing. Fig. C and Fig. D showed microbial composition at the genus level and species level among the four groups grouped by ethnicity and the mode of delivery (Han_Cesarean, Han_Vaginal, Li_Cesarean and Li_Vaginal). Species that represented less than 1% of all bacterial species were grouped in a “low abundance” category.

**Supplementary Table S1**. Multivariate redundance analyses showing association between ethnic, intrapartum antibiotics, mode of delivery, and other factors

| Multivariable | *p* | R^2^ | adjust R^2^ |
| --- | --- | --- | --- |
| Ethnic, Intrapartum antibiotics, mode of delivery | 0.266 | 0.187 | 0.16 |
| Ethnic, Intrapartum antibiotics, Lifestyle | 0.001*** | 0.743 | 0.735 |
| Ethnic, Intrapartum antibiotics, BMI | 0.081 | 0.895 | 0.652 |
| Ethnic, Intrapartum antibiotics, Age | 0.963 | 0.423 | 0.121 |
| Ethnic, Intrapartum antibiotics, Parity | 0.564 | 0.198 | 0.154 |
| Ethnic, mode of delivery, Lifestyle | 0.135 | 0.596 | 0.583 |
| Ethnic, mode of delivery, BMI | 0.792 | 0.938 | 0.752 |
| Ethnic, mode of delivery, Age | 0.456 | 0.486 | 0.178 |
| Ethnic, mode of delivery, Parity | 0.189 | 0.165 | 0.119 |
| Ethnic, Lifestyle, BMI | 0.018* | 0.934 | 0.783 |
| Ethnic, Lifestyle, Age | 0.044 | 0.776 | 0.647 |
| Ethnic, Lifestyle, Parity | 0.599 | 0.592 | 0.57 |
| Ethnic, BMI, Age | 0.001*** | 0.965 | 0.79 |
| Ethnic, BMI, Parity | 0.953 | 0.887 | 0.458 |

Redundancy values (R^2^) indicate the percent (%) variation explained by each individual factor (in univariate analyses) or each multivariable. **p* < 0.05, ***p* < 0.01, ****p* < 0.001.

**Supplementary Table. S2** Factors associated with colostrum microbiota α diversity
