# Supplementary figures and images for: Contrasting Diversity and Composition of Human Colostrum Microbiota in a Maternal Cohort With Different Ethnic Origins but Shared Physical Geography (Island Scale)
Source: Front Microbiol. 2022 Jul 12;13:934232. doi: 10.3389/fmicb.2022.934232 (PMC9315263; doi:10.3389/fmicb.2022.934232)

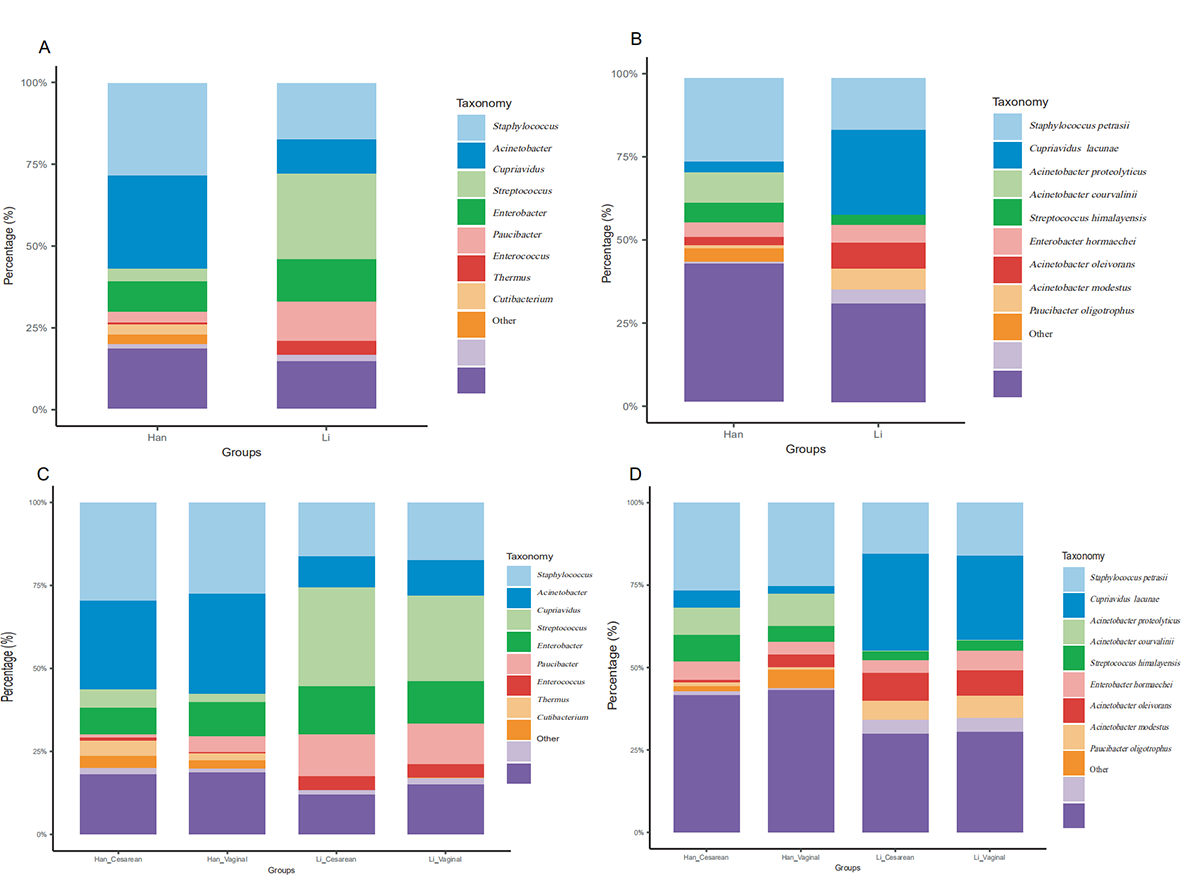

Supplement: Supplementary file 3 [file Image_1.TIF]
